# Sitagliptin eye drops prevent the impairment of retinal neurovascular unit in the new *Trpv2*^+/−^ rat model

**DOI:** 10.1186/s12974-024-03283-5

**Published:** 2024-11-30

**Authors:** Hugo Ramos, Josy Augustine, Burak M. Karan, Cristina Hernández, Alan W. Stitt, Tim M. Curtis, Rafael Simó

**Affiliations:** 1 Diabetes and Metabolism Research Unit, Vall d’Hebron Research Institute, Barcelona, 08035 Spain; 2 Centro de Investigación Biomédica en Red de Diabetes y Enfermedades Metabólicas Asociadas (CIBERDEM), Instituto de Salud Carlos III (ICSIII), Madrid, 28029 Spain; 3 Department of Medicine, Universitat Autònoma de Barcelona, Barcelona, 08193 Spain; 4 Wellcome-Wolfson Institute for Experimental Medicine, School of Medicine, Dentistry & Biomedical Science, Queen’s University Belfast, Belfast, Northern Ireland UK

**Keywords:** Diabetes, Diabetic retinopathy, Sitagliptin, DPP-4 inhibitor, Trpv2

## Abstract

Impaired function of the retinal neurovascular unit (NVU) is an early event in diabetic retinopathy (DR). It has been previously shown that topical delivery of the dipeptidyl peptidase-4 (DPP-4) inhibitor sitagliptin can protect against diabetes-mediated dysfunction of the retinal NVU in the *db/db* mouse. The aim of the present study was to examine whether sitagliptin could prevent the DR-like lesions within the NVU of the new non-diabetic model of DR, the *Trpv2* knockout rat (*Trpv2*^+/−^). For that purpose, at 3 months of age, *Trpv2*^+/−^ rats were topically treated twice daily for two weeks with sitagliptin or PBS-vehicle eyedrops. *Trpv2*^+/+^ rats treated with vehicle served as the control group. Body weight and glycemia were monitored. Optical coherence tomography recordings, fundus images and retinal samples were obtained to evaluate sitagliptin effects. The results revealed that sitagliptin eye drops had no effect on body weight or glycemia. Vehicle-treated *Trpv2*^+/−^ rats exhibited retinal thinning and larger diameters of major retinal blood vessels, upregulation of inflammatory factors and oxidative markers, glial activation and formation of acellular capillaries. However, topical administration of sitagliptin significantly prevented all these abnormalities. In conclusion, sitagliptin eye drops exert a protective effect against DR-like lesions in *Trpv2*^+/−^ rats. Our results suggest that sitagliptin eye drops carry significant potential to treat not only early-stages of DR but also other diseases with impairment of the NVU unrelated to diabetes.

## Introduction

Diabetic retinopathy (DR) is a common neurovascular complication of diabetes and remains a major cause of blindness worldwide [1]. This progressive disease disrupts normal retinal cell interactions within the neurovascular unit (NVU), leading to glial and neural dysfunction, as well as vascular abnormalities [2, 3]. Apart from recommending a tight control of blood glucose levels and hypertension, there are no well-established treatments for the early stages of DR and all current treatments are targeted at the late stages of the disease, when vision is often already affected. Additionally, these treatments are invasive, expensive, associated with severe side-effects and are not effective for all patients [4]. The increasing prevalence of diabetes and its associated burden on healthcare systems highlight the urgent need for early DR treatment. This necessitates the development of targeted therapies based on the pathophysiological events that take place in the early stages of this disease [4].

In the context of new experimental treatments against the early stages of DR, we have previously demonstrated that topical (eye drops) administration of agents that activate the glucagon-like peptide-1 (GLP-1) receptor (GLP-1R), whether directly or indirectly, prevents NVU impairment [5, 6]. These agents include native GLP-1 [5], GLP-1R agonists [7] and dipeptidyl peptidase-4 (DPP-4) inhibitors (DPP-4i) [6]. DPP-4i compounds, such as sitagliptin, prevent the rapid degradation of GLP-1 by DPP-4, which is present in the retinal pigment epithelium, thereby enhancing GLP-1 content in the retina [6]. GLP-1 is locally synthesized in the retina and GLP-1R activation plays a key physiological and neuroprotective role [5, 8]. The rationale for designing these new treatments is supported by the fact that GLP-1 production is downregulated in the diabetic retina [5]. Therefore, GLP-1 or DPP-4i eye drops could be considered as a replacement therapy to increase intraretinal GLP-1 levels. Notably, both GLP-1 and DPP-4i, particularly sitagliptin, besides neuroprotection, prevent vascular leakage in capillaries, a hallmark of microvasculature dysfunction [5, 6, 9]. Despite the effectiveness of these compounds, sitagliptin stands out due its comparatively lower cost, higher stability, and additional beneficial mechanisms [10–12]. This positions sitagliptin as a promising candidate for treating the early stages of DR. However, whether sitagliptin eye drops can prevent neurodegeneration and microvascular impairment in the absence of a diabetic *milieu* is unknown.

The therapeutic approach tested in the present study utilizes sitagliptin in eye drop solution form, which offers a more rational and safer route of administration compared to invasive intravitreal injections for treating the early stages of DR. This strategy allows self-administration by patients and reduces medical interventions and associated costs [13]. Furthermore, evidence suggests that systemic administration does not significantly impact DR beyond improving blood glucose levels [14–16]. This limitation may be attributed to the inability of most DPP-4i compounds to cross the blood-retinal barrier (BRB) [17, 18], a challenge that can be overcome by topical administration.

Recent reports indicate that heterozygous knockout rats for the *Trpv2* (Transient receptor potential cation channel subfamily V member 2) gene, despite being normoglycemic and normotensive, exhibit defects in the myogenic autoregulatory response in the retina which are similar to those observed in DR [19]. Furthermore, these autoregulatory deficits result in abnormal blood flow and associated glial activation, neurodegeneration, vascular leakage and vasodegeneration [19]. By reproducing one of the earliest events in the pathophysiology of the disease [20], the alterations in blood flow autoregulation, this model enables the evaluation of novel treatments targeting both early stages of DR and other retinal diseases involving NVU impairment. Specifically, in our study, the presence of DR-like lesions in the *Trpv2*^+/−^ model, which are not driven by hyperglycemia, provided us with the opportunity to explore whether sitagliptin eye drops could prevent neurodegeneration and microvascular damage in the NVU in a non-diabetic context. Our results provide evidence that topical administration of sitagliptin can prevent inflammation, oxidative stress, glial activation, retinal thinning, vessel dilatation and acellular capillary formation in the *Trpv2*^+/−^ rat. These findings suggest that the beneficial effects of sitagliptin eye drops can be extended to other models of NVU impairment not mediated by diabetes.

## Materials and methods

### Animals

Heterozygous *Trpv2*^*+/–*^ and wildtype *Trpv2*^*+/+*^ rats (Sprague Dawley background) were bred and maintained until 3 months of age for the study. Original breeders were generated from SAGE labs, as previously described [19], and both groups were obtained from heterozygous breeders as homozygous *Trpv*2^−/−^ animals are not viable. Rats were housed in the Biological Services Unit (BSU) at Queen’s University Belfast under SPF and normal lighting conditions (12-hour light/dark cycle). All in vivo experiments were approved by the Animal Welfare Ethical Review Body (AWERB) of Queen’s University Belfast. The protocol complied with the UK Home Office Animals (Scientific Procedures) Act 1986 (Project License, PPL2888) and the Association for Research in Vision and Ophthalmology (ARVO) statement for the use of animals in ophthalmology and vision research.

Body weights and blood glucose measurements (Alphatrak Glucometer; Zoetis, Leatherhead, UK) were obtained weekly. At the end of the experiment, body weights, blood glucose, and glycated hemoglobin (HbA1c) levels, measured with an A1CNOW^®^+ kit (PTS Diagnostics, Whitestown, IN, USA), were evaluated prior to animal euthanasia by CO_2_ asphyxiation.

### Topical ocular treatment

Topical administration of eye drops containing sitagliptin phosphate monohydrate (Selleckchem, Houston, TX, USA) at a concentration of 10 mg/mL, or vehicle [phosphate buffered saline (PBS)], was conducted twice daily for two weeks in 3-months old rats. The therapeutic regimen and sitagliptin dosage were selected based on our previous study on dose effectiveness in the db/db mouse model [9]. The age of the animals was chosen due to its close resemblance to the NVU pathology observed in humans with early DR. *Trpv2*^+/−^ rats aged 3 weeks did not exhibit any significant signs of damage, while animals aged 1 year showed a phenotype more similar to the later stages of the disease [19]. The animals were randomly allocated into one of three experimental groups: *(1) Trpv2*^*+/+*^ rats treated with vehicle; *(2) Trpv2*^*+/−*^ rats treated with vehicle; and *(3) Trpv2*^*+−+*^ rats treated with sitagliptin. Eye drops (15 µL) were randomly administered onto the superior corneal surfaces of both eyes of each animal using a pipette.

### Spectral-domain optical coherence tomography (SD-OCT)

Spectral-domain optical coherence tomography (SD-OCT) images (30° field of view) were acquired from all experimental groups after two weeks of sitagliptin eye drop treatment using a Spectralis Heidelberg OCT system (Heidelberg Engineering, Heidelberg, Germany). Rats were anesthetized using sodium pentobarbital (50 mg/kg; i.p) and pupils were dilated with 1% atropine sulphate (Bausch & Lomb, UK) and 2.5% phenylephrine hydrochloride (Bausch & Lomb, UK). Measurements were obtained using a standardized method to ensure consistent values from the same retinal locations across all animals. This method involved dividing the eye into four quadrants (superior, inferior, nasal, and temporal) and obtaining measurements of total retinal thickness, inner retinal thickness, and photoreceptor layer thickness in each quadrant. All measurements were consistently taken at an eccentricity of 1500 μm from the optic disc. The final value for each animal and for each individual layer or combination of layers was calculated by averaging the measurements across all quadrants [21].

### Retinal vessel caliber

Fundus images of the retina, captured with the optic nerve at the center, were taken from all experimental groups after two weeks of eye drop treatment using a Micron IV rat fundus camera (Phoenix Research Laboratories, OR, USA). Rats were anesthetized using sodium pentobarbital (50 mg/kg; i.p) and pupils were dilated with 1% atropine sulphate and 2.5% phenylephrine hydrochloride. Fundus images were processed and analysed using Automated Retinal Image Analyser (ARIA) v1.0 Software [16]. After defining the optic disk manually in the software, the arteriolar and venular diameters in a predefined region of interest (ROI) located at a distance ranging from one-and-a-half to two-and-a-half disc diameters from the optic disc were quantified. The four largest arterioles and venules in each image were selected for analysis, to ensure consistent vessel detection and precise calculation of average diameters for each vessel type within the ROI. Data from both eyes were averaged for each animal, and these values were subsequently used for statistical analysis.

### RNA extraction and quantitative reverse transcription polymerase chain reaction (RT-qPCR)

Total RNA from neuroretinas was isolated using the RNeasy Mini Kit (QIAGEN, Hilden, Germany). A Nanodrop spectrophotometer was used for the RNA quantification and 1000 nanograms of RNA were transcribed into cDNA using a high capacity cDNA Kit (ThermoFisher Scientific, Waltham, MA, USA), following the manufacturer’s guidelines. RT-qPCR was performed using a LightCycler 480 System (Roche Diagnostics, Basel, Switzerland) with SYBR Green PCR Master Mix (Roche Diagnostics, Basel, Switzerland) for specific primers (Table 1). Relative gene expression of transcripts was calculated using the comparative Ct method (2 − ΔΔCt) with data normalized to *Actb*. Data are displayed as fold change versus control *Trpv2*^*+/+*^ rats and a *n* of 4 retinas per experimental group was used.

**Table 1:** Primers used for RT-qPCR experiments

Primers	Gene ID	Sense	Nucleotide sequence
*Actb*	81,822	Forward (5’-3’)	5’ - ​TGCCCTAGACTTCGAGCAAG - 3’
		Reverse (5’-3’)	5’ -​ GGCAGCTCATAGCTCTTCTCC - 3’
*Aqp4*	25,293	Forward (5’-3’)	5’ - GCCACCTGGCTACAACCCTGG ​ - 3’
		Reverse (5’-3’)	5’ -​ CCATGGTGGCAATGCTGAGTCCA - 3’
*Ccl5*	81,780	Forward (5’-3’)	5’ -​ CCAATCTTGCAGTCGTGTTTG - 3’
		Reverse (5’-3’)	5’ -​ CATCTCCAAATAGTTGATGTA - 3’
*Glul*	24,957	Forward (5’-3’)	5’ - TGCCTGCCCAGTGGGAATT ​- 3’
		Reverse (5’-3’)	5’ -​ TATTGGAAGGGTTCGTCGCC - 3’
*Gsr*	116,686	Forward (5’-3’)	5’ -​ CCCTACCGTGGTCTTCAGCC - 3’
		Reverse (5’-3’)	5’ -​ CTTCCTCGTGGTCACAGCGT - 3’
*Icam1*	25,464	Forward (5’-3’)	5’ - CCTGGAGATGGAGAAGACCTTG ​- 3’
		Reverse (5’-3’)	5’ - GGGAAGTACCCTGTGAGGTG​ - 3’
*Il1b*	24,494	Forward (5’-3’)	5’ -​ CTCAATGGACAGAACATAAGCC - 3’
		Reverse (5’-3’)	5’ - GGTGTGCCGTCTTTCATCA ​- 3’
*Il16*	24,498	Forward (5’-3’)	5’ -​ GCCCTTCAGGAACAGCTATGA - 3’
		Reverse (5’-3’)	5’ - TGTCAACAACATCAGTCCCAAGA ​- 3’
*Il13*	116,553	Forward (5’-3’)	5’ - CATGGTATGGAGCGTGGACCT​ - 3’
		Reverse (5’-3’)	5’ - CTGGGTCCTGTGGATGGCATT - 3’
*Il18*	29,197	Forward (5’-3’)	5’ - GCACAGCCTCTCAGTTGGAAG ​- 3’
		Reverse (5’-3’)	5’ - GACTGCTGTACGTGGGTCCT ​- 3’
*Kcnj10*	29,718	Forward (5’-3’)	5’ -​ ACTACCCCAGGATTCATCAGAGCA - 3’
		Reverse (5’-3’)	5’ -​ GCCTGTAAAGGTTGGCGAGAA - 3’
*Nrf2*	83,619	Forward (5’-3’)	5’ - CTCTCTGGAGACGGCCATGACT ​- 3’
		Reverse (5’-3’)	5’ -​ CTGGGCTGGGGACAGTGGTAGT - 3’
*Ppb7*	246,358	Forward (5’-3’)	5’ - CGTAACCTACAGGTGCTGCT ​- 3’
		Reverse (5’-3’)	5’ - TCCTGGCCGGAACACATTC​ - 3’
*Sod1*	24,786	Forward (5’-3’)	5’ -​ AATGTGTCCATTGAAGATCGTGTGA - 3’
		Reverse (5’-3’)	5’ -​ GCTTCCAGCATTTCCAGTCTTTGTA - 3’
*Sod2*	24,787	Forward (5’-3’)	5’ - AGGGCCTGTCCCATGATGTC ​ - 3’
		Reverse (5’-3’)	5’ -​ AGAAACCCGTTTGCCTCTACTGAA - 3’
*Tnfa*	24,835	Forward (5’-3’)	5’ -​ GTCGTAGCAAACCACCAAGC - 3’
		Reverse (5’-3’)	5’ -​ TGTGGGTGAGGAGCACGTAG - 3’
*Trpv2*	29,465	Forward (5’-3’)	5’ - GACCTCCTAAAAACACTTCTGCTC - 3’
		Reverse (5’-3’)	5’ -​ AGAGTCGGTCACGGTCAAAC - 3’
*Vegfa*	83,785	Forward (5’-3’)	5’ - GCAGAAAGCCCATGAAGTGGTG​ - 3’
		Reverse (5’-3’)	5’ -​ TTCATCATTGCAGCAGCCCG - 3’

### Immunohistochemistry of rat retinal frozen sections and whole mount preparations

Ocular globes were collected and fixed in 4% paraformaldehyde for one hour at room temperature. After 3 days of 30% sucrose treatment, the eyes were cryosectioned to a thickness of 14 μm and stored at -20 °C. For immunohistochemistry experiments, frozen sections were washed, blocked and incubated overnight with primary antibodies (Table 2) at 4 °C. The next day, the samples were washed for 3 × 15 min and were incubated with secondary antibodies (Table 2) for one hour at room temperature. The samples were then washed for 4 × 15 min and were mounted with VECTASHIELD antifade mounting medium supplemented with DAPI for nuclei staining (Vector Labs, Burlingame, CA). Z-stack images (step size of 2 μm) were obtained with a Leica SP8 confocal microscopy (Leica Biosystems, Wetzlar, Germany).

Rat retinal whole mounts were enucleated from the eyes fixed in 4% paraformaldehyde for one hour at room temperature and stored in methanol for at least 3 days at -20 °C. For immunohistochemistry experiments, retinas were washed, blocked, and incubated for 3 days with primary antibodies at 4 °C (Table 2). After 3 days, the samples were washed for 8 × 30 min and were incubated with secondary antibodies (Table 2) at 4 °C. The samples were then washed for 8 × 30 min. For the detection of isolectin binding, isolectin-B4 from *Bandeiraea simplicifolia* (1:200; biotin conjugate; Sigma-Aldrich; catalog L2140) was used, followed by incubation with streptavidin conjugated to Alexa Fluor 647 (1:200; Invitrogen; catalog S21374). The samples were then whole-mounted (eyecup facing up and the retina receives four incisions to form a Maltese cross) with VECTASHIELD antifade mounting medium (Vector Labs). Z-stack images (step size of 2 μm) of stained whole-mounts were acquired with a Leica SP8 confocal microscopy (Leica Biosystems, Wetzlar, Germany).

**Table 2:** Primary and secondary antibodies used for immunohistochemistry

**Primary antibodies**	**Dilution**	**Company**	**Catalogue number**
8-hydroxyguanosine	1:100	Abcam	AB62623
Collagen IV	1:75	Bio-rad	2150 − 1470
GFAP	1:200	DAKO	Z0334
IBA1	1:200	Invitrogen	PA5-18039
**Secondary antibodies**	**Dilution**	**Company**	**Description**
Donkey anti-mouse Alexa Fluor 488	1:300	Invitrogen	A32766
Donkey anti-Rabbit Alexa Fluor 488	1:300	Invitrogen	A21206
Donkey anti-Rabbit Alexa Fluor 568	1:300	Invitrogen	A10042
Donkey anti-mouse Alexa Fluor 568	1:300	Invitrogen	A10037
Donkey anti-goat Alexa Fluor 405	1:300	Invitrogen	A48259

#### Analysis of images

The parameters used for confocal imaging were kept consistent across slides and the images were processed using FIJI software. For markers evaluated on stained retinal cryosections, an *n* of 4 retinas from 4 different animals per experimental group was used. 3 x Z-stack images were captured per retinal section and merged prior to quantification. GFAP quantification entailed measurement of the number of GFAP positive fibers per 100 μm of retinal width, while for IL-33 the number of positive cells per mm^2^ was counted. Immunofluorescence staining with 8-hydroxyguanosine was assessed by measuring its relative expression across all layers of the retina.

For those markers studied with whole-mount preparations (IBA-1, Collagen IV and Isolectin B4), an *n* of 4 retinas from 4 different animals per experimental group was used. 8 x Z-stack images were captured per retina, randomly selecting both central and peripheral areas. Z-stack images were merged prior to quantification. For the study of microglia, each Z-stack was segmented into three parts corresponding to ganglion cell layer (GCL), inner plexiform layer (IPL) and outer plexiform layer (OPL) of the retina, and the number of cells per mm^2^ was quantified. For colabelling with Isolectin B4 and Collagen IV, the number of acellular capillaries per mm^2^ was counted. Acellular capillaries were identified as those positive for Collagen IV staining but negative for Isolectin B4 staining.

### Statistics

Data is displayed as mean ± SEM. Statistical analysis was conducted using Prism 6 (GraphPad Software, CA, USA). Normality and equal variance were assessed before applying one-way analysis of variance (ANOVA) or two-way ANOVA with suitable post hoc tests. A significance level of *p* < 0.05 was considered statistically significant. No outliers were detected upon evaluation using Grubb’s test.

## Results

### Physiological parameters of experimental groups

**Fig. 1:**
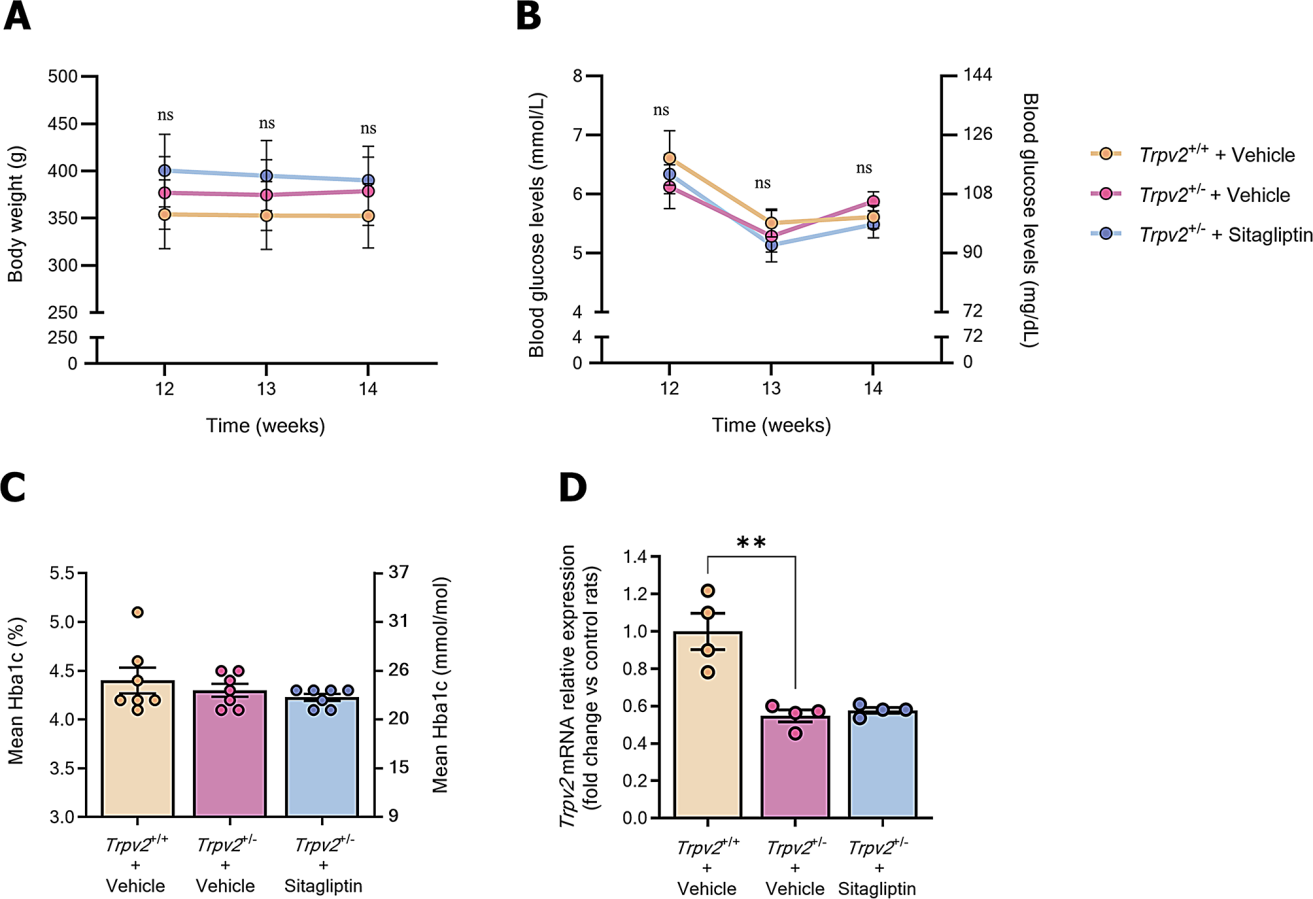
Evaluation of physiological parameters. **A**,**B** Body weight **A** glycaemic **B** measurements during the experimental course of *Trpv2*^+/−^ rats treated with sitagliptin (blue) or vehicle (purple) and *Trpv2*^+/+^ wild type rats treated with vehicle (cream-colour); *n* = 7. **C** Bar graph illustrating the average HbA1c measurements per each group at the end of the experiment, prior to euthanasia. Control homozygous rats are represented with a green bar, while vehicle-treated heterozygous rats are represented with a red bar and sitagliptin-treated heterozygous rats with a blue bar; *n* = 7. **D** Bar graph showing mean values from the RT-qPCR analysis of *Trpv2* gene in vehicle-treated *Trpv2*^+/+^ rats (cream-colour) and in *Trpv2*^+/−^ heterozygous rats that received vehicle (purple) or sitagliptin eye drops (blue). Results are presented as fold change vs. control mice; *n* = 4. ns = no significance, ** *p* < 0.01

We did not observe any differences in body weight, glycemia and HbA1c levels among the experimental groups at baseline or throughout the experimental course (Fig. 1A-C).

To confirm the heterozygous condition of *Trpv2*^+/−^ rats, *Trpv2* mRNA levels were evaluated in the retinas. The results showed lower *Trpv2* expression in heterozygous animals (Fig. 1D). Additionally, sitagliptin eye drops did not impact the gene expression of TRPV2 channels (Fig. 1D). *n* = 7. * *p* < 0.05

### Topical administration of sitagliptin prevents retinal thinning in *Trpv2*^+/−^ rats

**Fig. 2:**
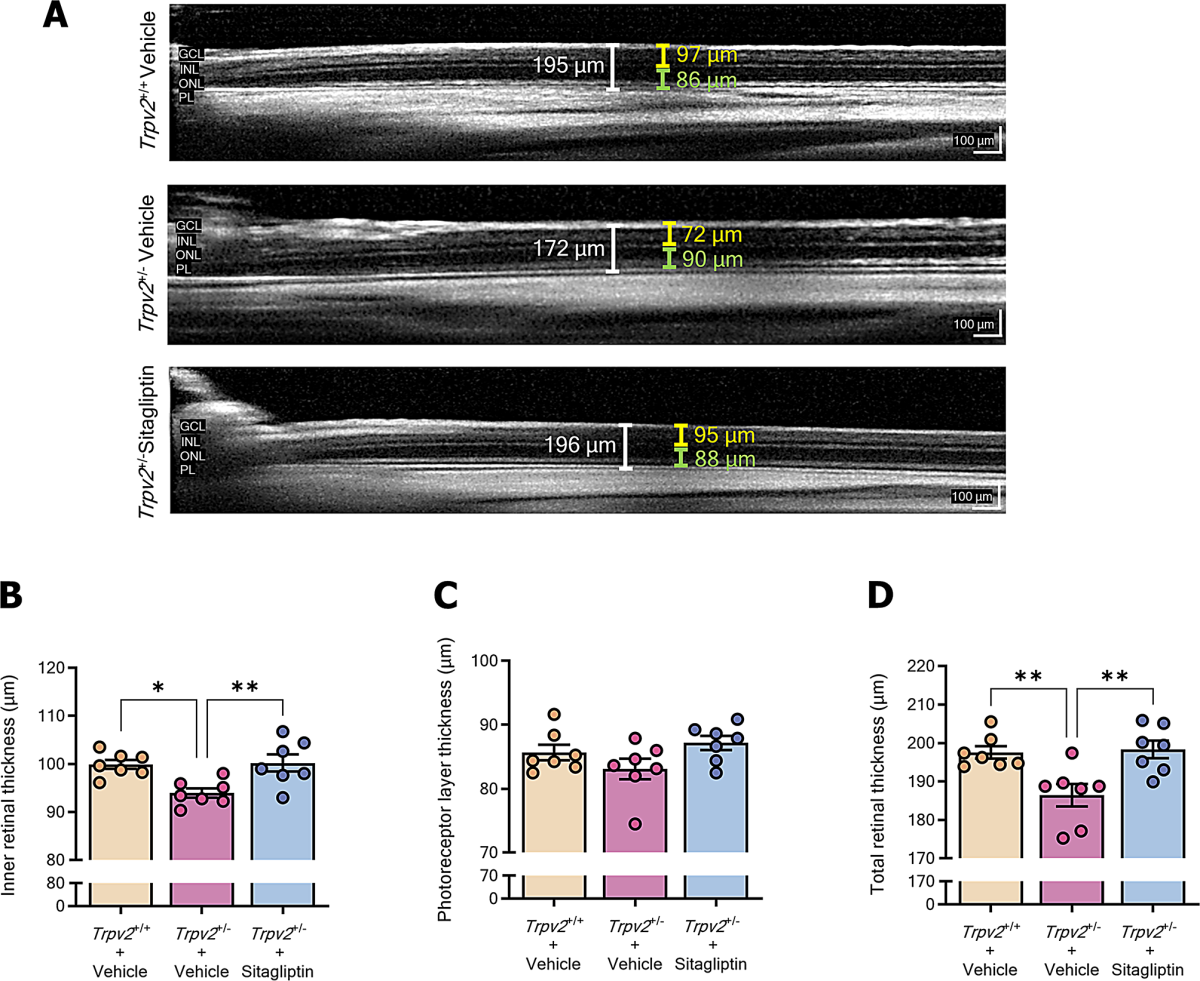
Retinal thickness assessment through SD-OCT. Measurements were obtained at a consistent eccentricity of 1500 μm from the optic disc in four quadrants (superior, inferior, nasal, and temporal) of the eye. Total retinal thickness, inner retinal thickness, and photoreceptor layer thickness were measured in each quadrant, and the final value was calculated as the mean across all four quadrants. **A** Representative images of SD-OCT thickness measurements of inner retina (yellow bar), photoreceptor layer (green bar), and total neuroretina from *Trpv2*^+/−^ rats treated with vehicle or sitagliptin and wild-type rats treated with vehicle. Scale bar: 100 μm. **B-D** Bar graphs showing the mean values for the thickness measurements of inner retina **B**, photoreceptor layer **C** and total retina **D**. Control homozygous rats are represented with a cream-colored bar, while vehicle-treated heterozygous rats are represented with a purple bar and sitagliptin-treated heterozygous rats with a blue bar; *n* = 7. * *p* < 0.05, ** *p* < 0.01

Previous results describing the DR-like lesions in the *Trpv2*^*+/−*^ rat indicated that the neurodegenerative component of the disease is well represented in this model, with neurons in all retinal layers exhibiting some degree of cell death [19]. To evaluate the impact of this neuronal death on the inner retina, the photoreceptor layer, and total retinal thickness, as well as the potential effect of sitagliptin eye drops, SD-OCT images were captured. Retinal thinning was observed in the inner and total retinal layers of 3 months-old *Trpv2*^+/−^ rats, but not in the photoreceptor layer (Fig. 2A-D). Topical administration of sitagliptin prevented the retinal thinning observed in *Trpv2*^+/−^ rats (Fig. 2A-D).

### Sitagliptin eye drops prevent abnormal vasodilation in *Trpv2*^+/−^ rats

**Fig. 3:**
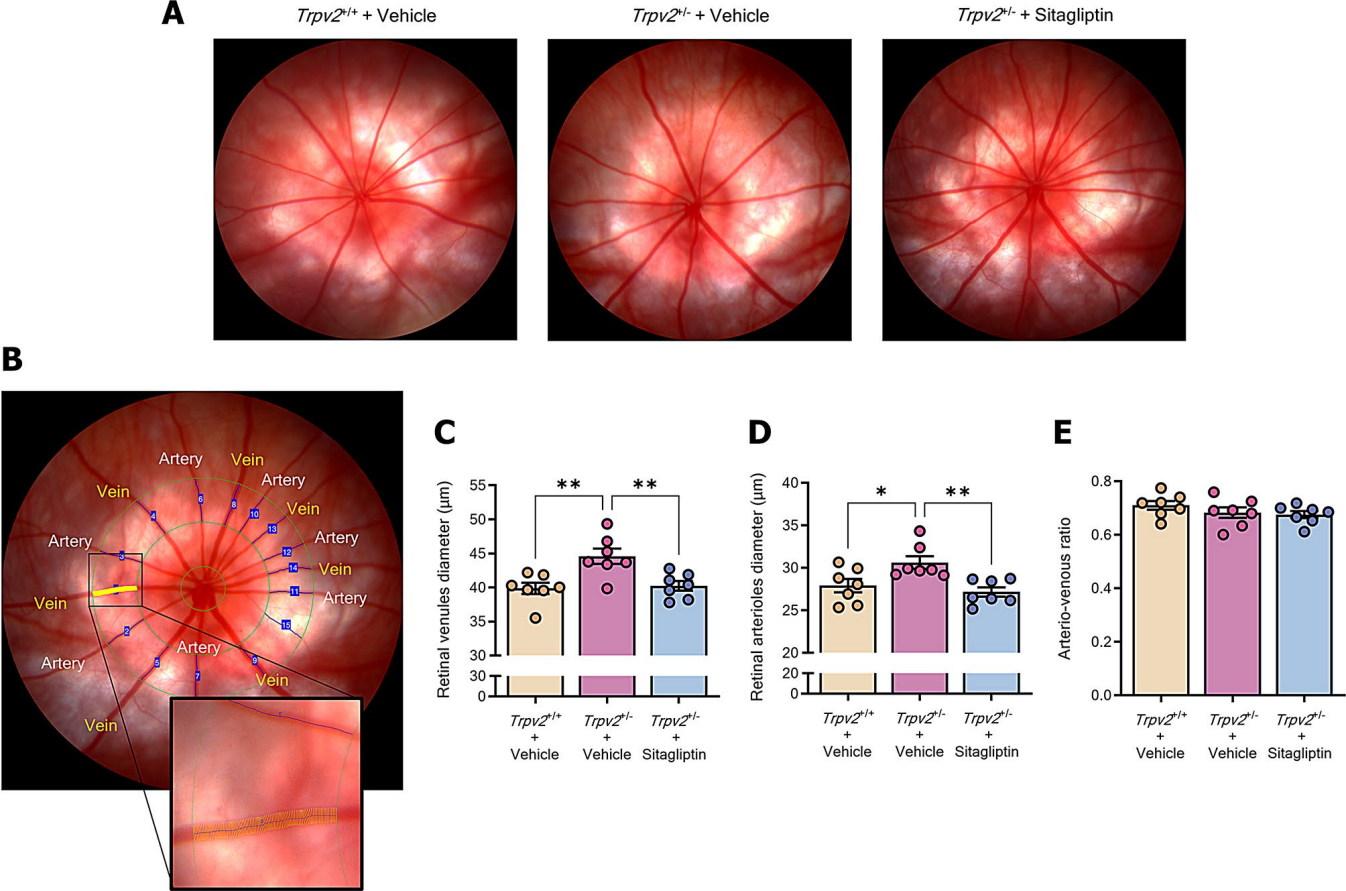
Funduscopic examination and diameter measurements of major retinal vessels. Fundus images were captured with the optic nerve at the centre and analysed using ARIA software, with the optic disc manually defined. Arteriolar and venular diameters were measured in a predefined region located at a distance ranging from one-and-a-half to two-and-a-half disc diameters from the optic disc. The four largest arterioles and venules were selected for consistent analysis, and data from both eyes were averaged per animal for statistical evaluation. **A** Representative fundus images of all the studied experimental groups. **B** Exemplifying images of the procedure of diameter analysis for major veins and arteries of the retina. The small green circle represents the optic disc, while the medium and large green circles delimit the studied area for all vessels. The yellow labelling illustrates the vessel under analysis at that time, while the enlarged image shows each of the measurements with thin yellow lines. **C**-**E** Bar graphs depicting the mean values of diameter measurements for main retinal veins **C** and arteries **D** and arterio-venous ratio **E** among experimental groups. Control homozygous rats are represented with cream-colored bars, while vehicle-treated heterozygous rats are represented with purple bars and sitagliptin-treated heterozygous rats with blue bars; *n* = 7. * *p* < 0.05, ** *p* < 0.01

Retinal arteriolar dilation serves as an indicator of loss of retinal blood flow autoregulation, which is a feature of the early stages of DR. Fundus images from experimental groups showed no visible signs of DR-like damage, such as microaneurysms, haemorrhages, exudates, or cotton wool spots (Fig. 3A). Nonetheless, ARIA vessel analysis software (Fig. 3B) revealed abnormal vasodilatation of major retinal vessels in the heterozygous rats treated with vehicle when compared to *Trpv2*^*+/+*^ wild-type rats treated with vehicle (Fig. 3C and D). Topical administration of sitagliptin prevented this abnormality (Fig. 3C and D). Due to the dilation of both the retinal arterioles and venules in heterozygous rats treated with vehicle, no significant change in the arterio-venous ratio was observed across the groups (Fig. 3E).

### Sitagliptin eye drops reduces vasodegeneration in *Trpv2*^+/−^ rats

**Fig. 4:**
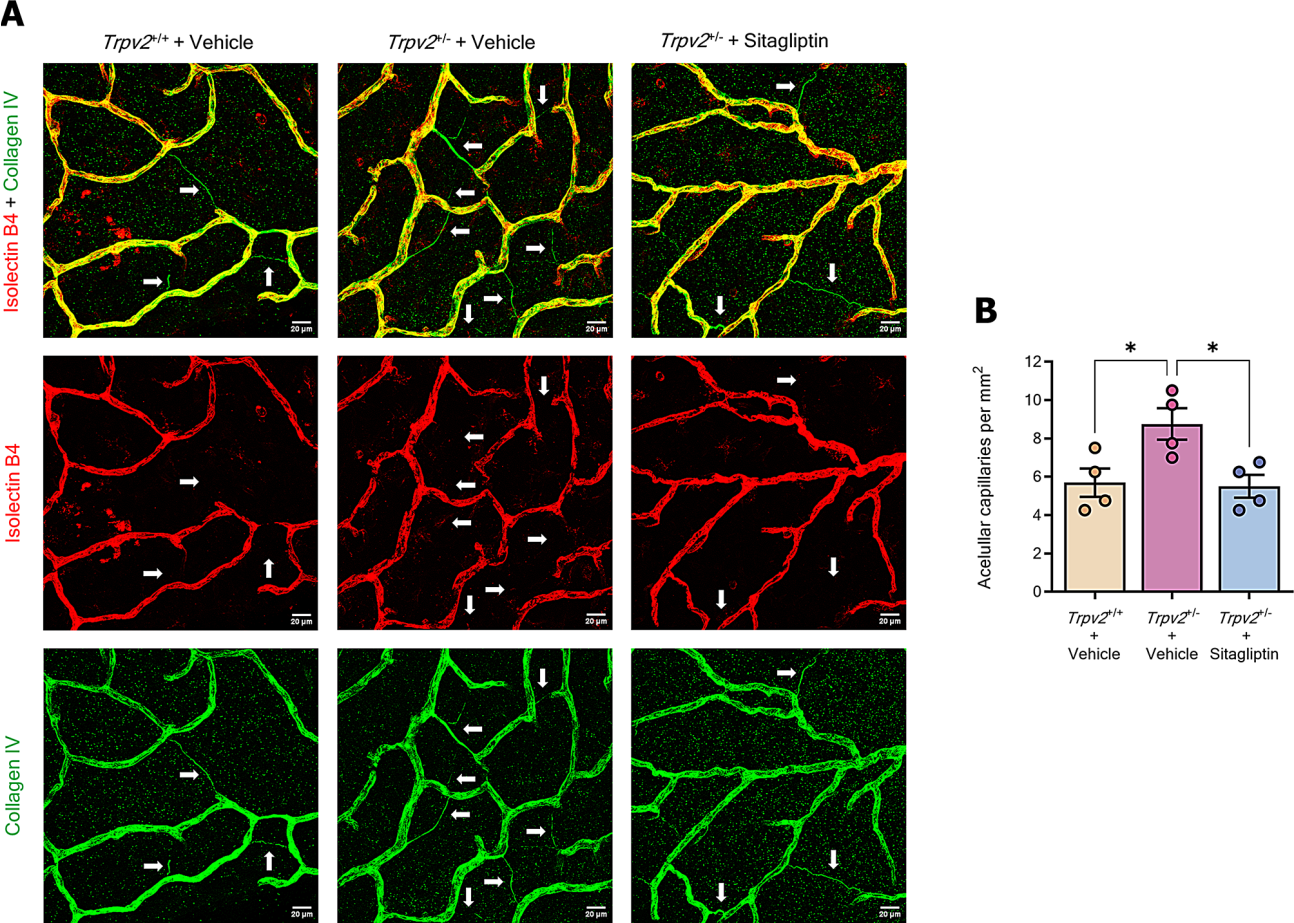
Analysis of acellular capillary formation. **A** Representative confocal Z-stack images of retinal whole mount preparations stained with collagen IV (green) and isolectin-B4 (red) focused at the superficial vascular plexus. Acellular capillaries, indicated with white arrows, are identifiable as deteriorated vascular segments showing positivity for collagen IV but negativity for isolectin-B4. Scale bars: 20 μm. **B** Bar graph illustrating the average number of acellular capillaries per experimental group. Control homozygous rats are represented with a cream-colored bar, while heterozygous rats treated with vehicle or sitagliptin are represented with a purple bar and a blue bar respectively. *n* = 4. * *p* < 0.05

In addition to retinal arteriolar dilation, the loss of endothelial cells of capillaries is a hallmark feature of DR. These acellular capillaries can appear as empty basement membrane tubes or more frequently, as coarse string remnants [22]. The retinas of *Trpv2*^+/−^ rats treated with vehicle exhibited higher numbers of acellular capillaries in the superficial layer of blood vessels, compared to wild-type rats. Topical administration of sitagliptin prevented the loss of capillaries in *Trpv2*^+/−^ rats, significantly reducing the formation of acellular capillaries (Fig. 4A and B).

### Müller cell activation is attenuated by sitagliptin eye drops in *Trpv2*^+/−^ rats

**Fig. 5:**
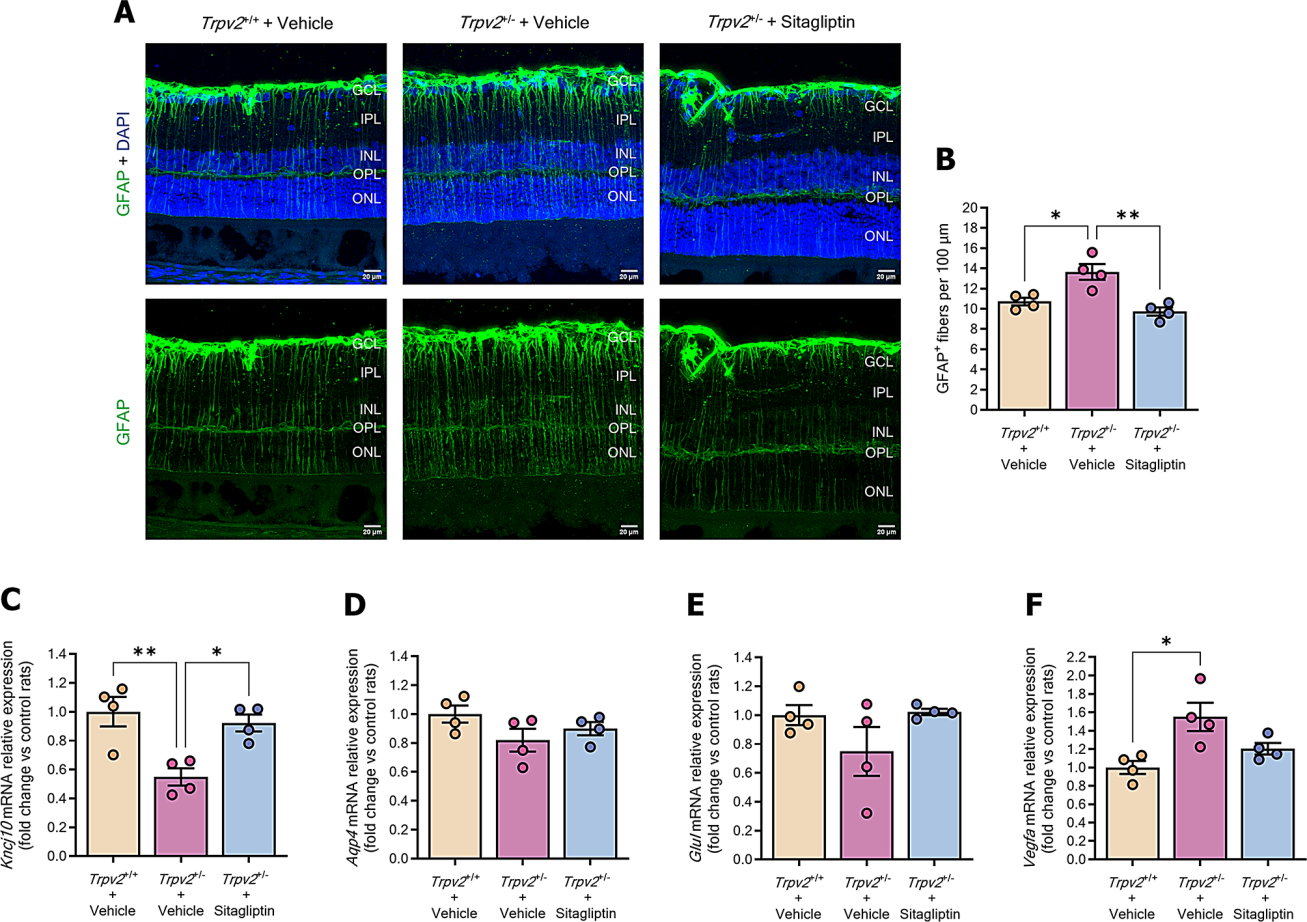
Evaluation of Müller cell activation. **A** Representative confocal Z-stack images of retinal criosections stained with GFAP (green) and DAPI (blue) from each experimental group. Scale bars: 20 μm. **B** Bar graph with the average numbers of GFAP + fibres located across the whole retina. Control *Trpv2*^+/+^ rats are represented with cream-colored bars, while vehicle-treated *Trpv2*^+/−^ rats are represented with red bars and sitagliptin-treated *Trpv2*^+/−^ rats with blue bars; *n* = 4. * *p* < 0.05, ** *p* < 0.01. **C-F** Bar graphs illustrating mean values from the RT-qPCR analysis of *Kncj10* **C**, *Aqp4* **D**, *Glul* **E** and *Vegfa* **F** genes in vehicle-treated *Trpv2*^+/+^ homozygous rats (cream-colour) and in *Trpv2*^+/−^ heterozygous rats that received vehicle (purple) or sitagliptin eye drops (blue). Results are presented as fold change vs. control mice; *n* = 4. GCL (ganglion cell layer), IPL (inner plexiform layer), INL (inner nuclear layer), OPL (outer plexiform layer), ONL (outer nuclear layer). * *p* < 0.05, ** *p* < 0.01

Early in DR, the homeostatic function of Müller cells is disrupted in response to stimuli such as oxidative stress and inflammation. Müller cell activation involves biochemical, physiological and structural changes, which are marked by the upregulation of glial fibrillary acidic protein (GFAP) expression [23]. We observed overexpression and increased GFAP content in the retinas of *Trpv2*^+/−^ rats treated with vehicle eye drops compared to the wild-type control group (Fig. 5A and B), demonstrating the presence of glial activation in this model. Topical administration of sitagliptin was effective in preventing the appearance of glial activation (Fig. 5A and B). In addition, we evaluated different key elements related to the physiological functions of Müller cells. Retinas from vehicle-treated *Trpv2*^+/−^ rats exhibited reduced mRNA levels of the inwardly rectifying potassium channel 4.1 (Kir 4.1) (*Kncj10* gene) (Fig. 5C), similar to what occurs in diabetic conditions [12]. However, no significant alteration was detected in the levels of aquaporin 4 (Aqp4) channel (Fig. 5D) and glutamine synthetase (Fig. 5E). Sitagliptin eye drops prevented the deleterious effect on Kir.4.1 expression (Fig. 5C).

Müller cells are a primary source of VEGF (vascular endothelial growth factor) during DR, a pro-angiogenic factor that contributes to retinal vascular leakage and neovascularization [24]. Our results revealed that the retinas of the *Trpv2*^+/−^ model exhibited increased expression of VEGF mRNA. Sitagliptin eye drops appeared to preserve physiological VEGF levels observed in control rats (Fig. 5F).

### Topical administration of sitagliptin reduces the number of microglial cells in the retina of *Trpv2*^+/−^ rats

**Fig. 6:**
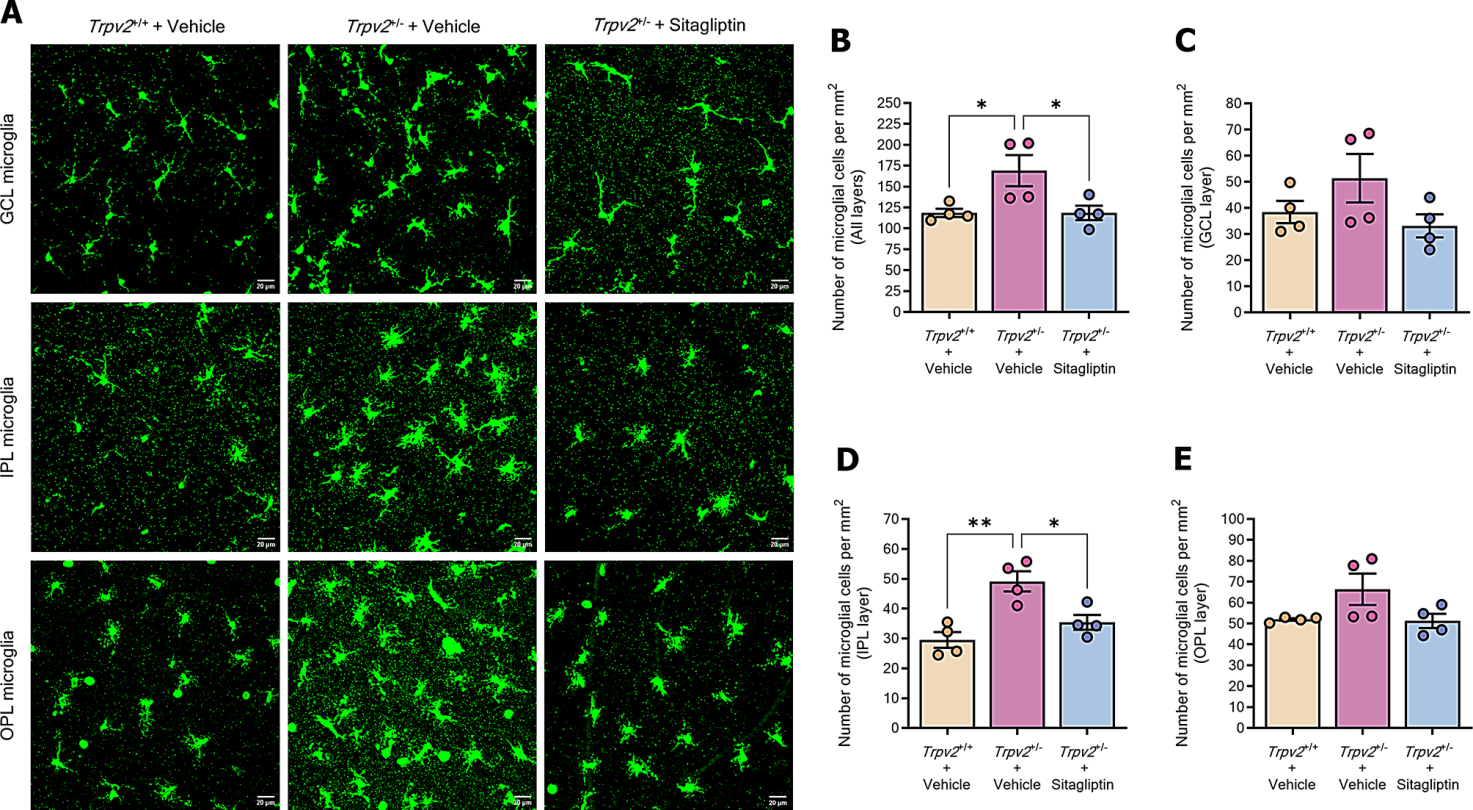
Assessment of number of microglial cells. **A** Representative confocal Z-stack images of whole-mount preparations labelled with IBA-1 (green) from each experimental group. Scale bars: 20 μm. **B**-**E** Bar graphs depicting the average number of microglial cells per mm^2^ of GCL **B**, IPL **c**, OPL **D** and all **E**)microglial layers of the retina for each studied experimental group. Control homozygous rats are represented with a cream-colored bar, while heterozygous rats treated with vehicle or sitagliptin are represented with a purple bar and a blue bar respectively. *n* = 4. GCL (ganglion cell layer), IPL (inner plexiform layer), OPL (outer plexiform layer). * *p* < 0.05, ** *p* < 0.01

Microglia are highly specialized phagocytic cells, that constantly monitor local synaptic activity and regulate the clearance of dying cells and metabolic debris in the retina [25]. Upon activation, microglia start to proliferate and undergo morphological (ramified to amoeboid) and functional changes, enhancing their immunoreactivity and migratory properties [26]. Here we evaluated the number of microglial cells per mm^2^ across the three experimental groups. The analysis included counts from the entire retina and from three distinct locations: ganglion cell layer (GCL), inner plexiform layer (IPL) and outer plexiform layer (OPL) [27, 28]. *Trpv2*^+/−^ heterozygous rats exhibited higher total numbers of microglial cells per mm^2^ in the retina compared to control wild-type rats (Fig. 6A and B). Individual layer evaluation revealed that these differences were mainly located in the IPL microglial layer, with no differences observed in the GCL and OPL layers (Fig. 6A, C and E). Sitagliptin eye drops prevented the increased levels of microglial cells in the IPL and total layers observed in *Trpv2*^+/−^ heterozygous rats, while showing similar values to wild-type rats in the GCL and OPL layers, (Fig. 6A-E).

### Sitagliptin eye drops decreases the expression of pro-inflammatory cytokines in *Trpv2*^+/−^ rats

**Fig. 7:**
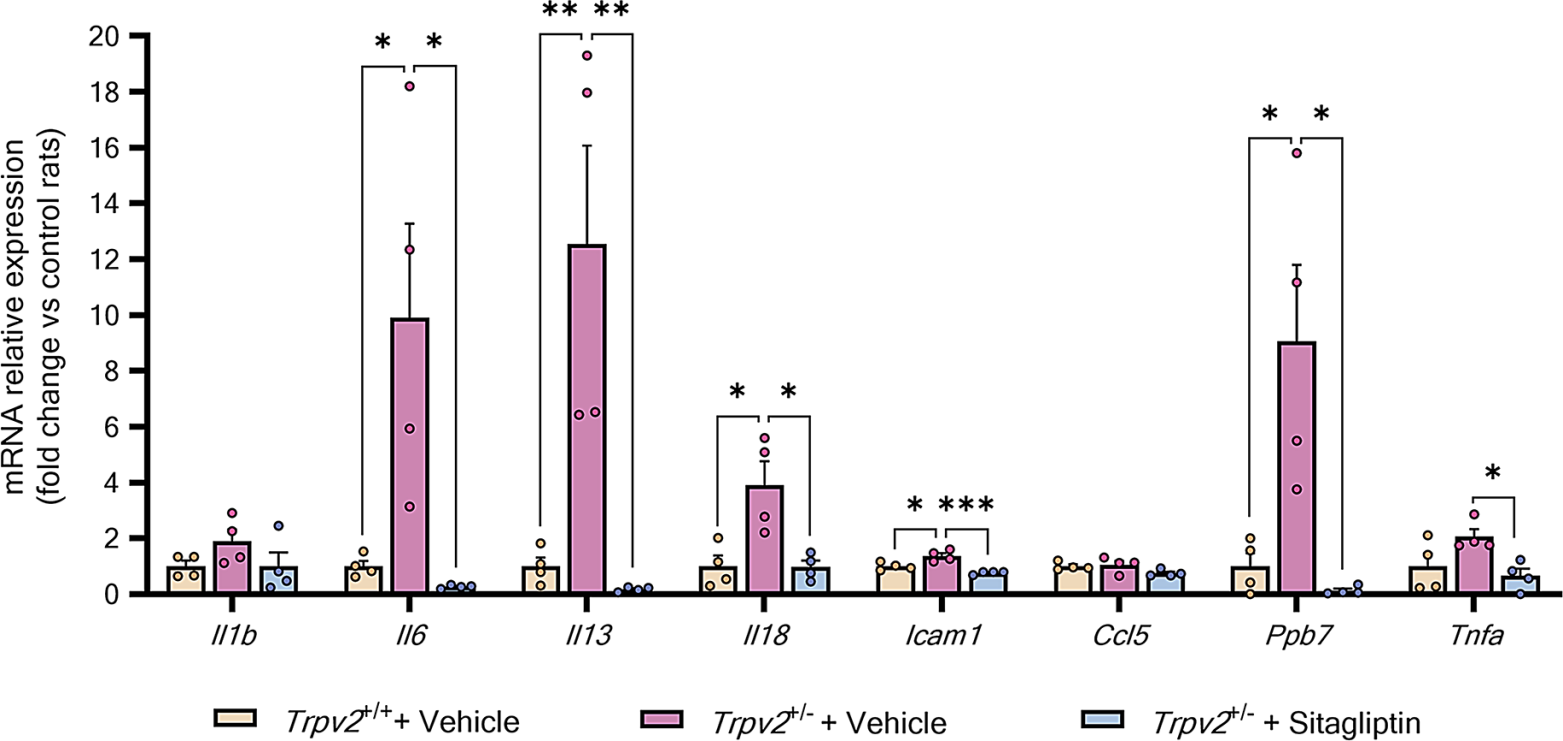
Evaluation of the pro-inflammatory environment. **A** Bar graph showing the mean values from the RT-qPCR analysis of genes that codify for some pro-inflammatory cytokines in vehicle-treated *Trpv2*^+/+^ homozygous rats (cream-colour) and in *Trpv2*^+/−^ heterozygous rats that received vehicle (purple) or sitagliptin eye drops (blue). Results are presented as fold change vs. control mice; *n* = 4. * *p* < 0.05, ** *p* < 0.01, *** *p* < 0.001

Previous findings with *Trpv2*^+/−^ rats demonstrated higher expression of multiple cytokines at 3 months of age compared to *Trpv2* wild-type rats [19]. In this study, we evaluated the effect of topical administration of sitagliptin on the expression of several cytokines, the intercellular adhesion molecule-1 (*Icam1*) and the chemokine (C-X-C motif) ligand 7 (*Ppbp*). We found significantly higher mRNA levels of interleukin-6 (*Il6*), interleukin-13 (*Il13*), interleukin-18 (*Il18*), *Icam1* and *Ppbp* in the retinas of heterozygous rats when compared to wild-type control rats (Fig. 7). However, there was no significant increase in interleukin 1 beta (*Il1b*) and C-C Motif chemokine ligand 5 (*Ccl5*), and only a non-significant trend was observed for tumor necrosis factor alpha (*Tnfa*) (Fig. 7). Sitagliptin eye drops significantly reduced the mRNA levels of *Il6*, *Il13*, *Il18*, *Icam1*, *Ppbp*, and *Tnfa* in the retinas of Trpv2^+/−^ rats (Fig. 7), while *Il1b* and *Ccl5* levels remained unaffected. (Fig. 7).

### Sitagliptin eye drops protect against oxidative stress by enhancing key antioxidant mechanisms

**Fig. 8:**
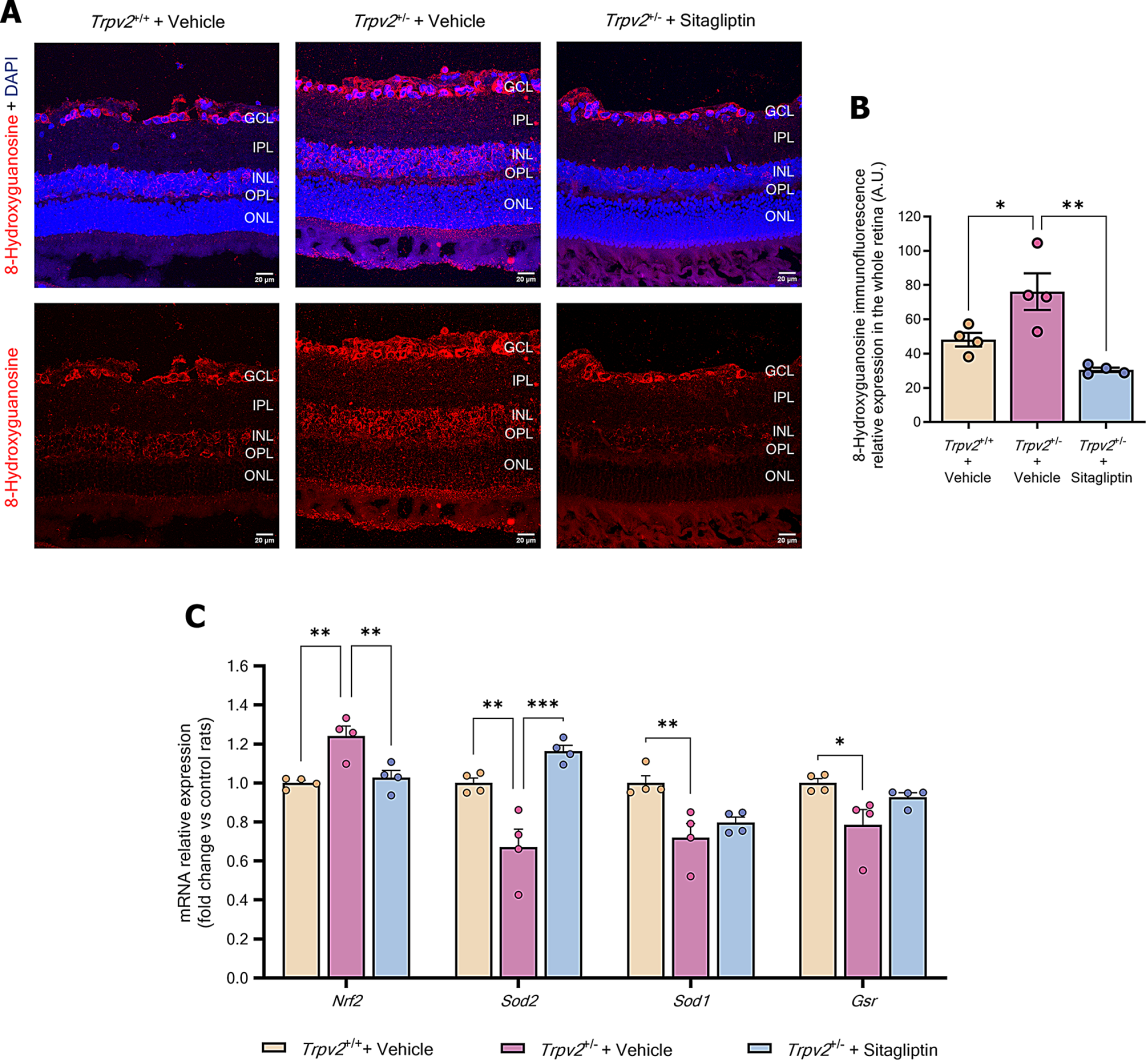
Examination of DNA oxidative damage and retinal antioxidant mechanisms. **A** Representative confocal Z-stack images of 8-hydroxyguanosine (red) and DAPI (blue) stainings on retinal criosections. **B** Bar graph illustrating the mean values of 8-hydroxyguanosine immunofluorescence intensity of each experimental group. Control homozygous rats are represented with cream-colored bars, while vehicle-treated heterozygous rats are represented with purple bars and sitagliptin-treated heterozygous rats with blue bars; *n* = 4. **c** Bar graph illustrating mean values from the RT-qPCR analysis of *Nrf2*, *Sod1*, *Sod2* and *Gsr* genes in vehicle-treated *Trpv2*^+/+^ homozygous rats (cream-colour) and in *Trpv2*^+/−^ heterozygous rats that received vehicle (purple) or sitagliptin eye drops (blue). Results are presented as fold change vs. control mice; *n* = 4. GCL (ganglion cell layer), IPL (inner plexiform layer), INL (inner nuclear layer), OPL (outer plexiform layer), ONL (outer nuclear layer). * *p* < 0.05, ** *p* < 0.01, *** *p* < 0.001

The oxidative status of the retina was assessed by measuring the levels of 8-hydroxyguanosine, one of the main products of DNA/RNA damage [29], and key antioxidant defences including the nuclear factor erythroid 2–related factor 2 (*Nrf2*) and some of its downstream targets: superoxide dismutase 1 and 2 (*Sod1* and *Sod2*) and glutathione reductase (*Gsr*). Heterozygous rats displayed higher levels of DNA/RNA oxidative damage compared to wild-type animals (Fig. 8A-B). Despite upregulation of *Nrf2*, mRNA levels of antioxidant enzymes were significantly reduced in Trpv2^+/−^ rats (Fig. 8C). Administration of sitagliptin eye drops partially prevented these changes (Fig. 8C). Importantly, sitagliptin eye drops improved the oxidative balance as evidenced by reduced levels of 8-hydroxyguanosine, indicating decreased DNA/RNA oxidation (Fig. 8A-B).

## Discussion

Recent advances in our understanding of the pathogenesis of DR have led to the design of new experimental treatments which address early-stage disease. Although the sequence of some pathological events during early DR is not entirely understood, it is established that neurodegeneration plays an important role in a large percentage of patients [30]. Therefore, it could be hypothesized that neuroprotective therapies might be effective, and there is evidence supporting this hypothesis [31–33]. Within current experimental approaches aiming to achieve neuroprotection, therapeutic interventions involving GLP-1R activation and, more recently, DPP-4 inhibitors, have demonstrated promising experimental results to address this unmet medical need [5, 6, 9–12, 34].

In the present study, we tested the effect of topical administration of sitagliptin (a DPP-4i) in 3-month-old *Trpv2*^+/−^ rats, a new non-diabetic model that replicates the NVU lesions observed in early stages of DR due to impaired retinal blood flow autoregulation [19]. We found that sitagliptin eye drops prevented all the pathological hallmarks shared with DR in *Trpv2*^+/−^ rats, including retinal thinning, glial activation, inflammation, oxidative stress, and abnormal and sustained vasodilatation. Notably, these beneficial effects were observed without any impact on systemic parameters such as body weight or glycemia, and they cannot be attributed to a possible restoration of the levels of TRPV2 channels mediated by sitagliptin.

We found that *Trpv2*^+/−^ rats exhibit retinal thinning similar to that seen in DR [34], a progressive event resulting from neurodegenerative mechanisms that affect the retina [35, 36]. Topical administration of sitagliptin prevented this pathological thinning after two weeks of treatment. Since emerging evidence suggests that retinal thinning precedes the onset of DR [37–40], this result reinforces previous studies indicating that neuroprotective agents can prevent this early hallmark of the disease [41]. This beneficial effect on *Trpv2*^+/−^ rats could be explained by the neuroprotective properties that we have previously reported in the *db/db* mouse model, where sitagliptin demonstrated efficacy against oxidative stress [12], inflammation and synaptic damage [10–12], thus contributing to restore physiological rates of neurogenesis [9, 12].

Glial activation plays a central role in the NVU dysfunction that occurs in DR, linking the neurodegenerative process with microvascular impairment. In the present study, as previously demonstrated in *db/db* mice [6], we found that sitagliptin eye drops prevented glial activation in *Trpv2*^+/−^ rats. This was evidenced by the abrogation of aberrant GFAP overexpression in this non-diabetic model. Consequently, the drug also protected against the downregulation of the Kir4.1 channel and the upregulation of VEGF. In addition, topical sitagliptin prevented the abnormal accumulation of microglial cells, mainly in the IPL layer of the retina, in *Trpv2*^+/−^ rats. Glial cell activation is crucial in the inflammatory process that occurs in diabetic retina, and we report that sitagliptin prevented the upregulation of several pro-inflammatory cytokines, including IL-6, IL-13, IL-18, ICAM-1, CXCL7 and TNF-α. These results are consistent with our previous study using *db/db* mice [12] and corroborated by in vitro studies [34, 42].

Both glial activation and inflammation play significant roles in the initial oxidative environment observed in the diabetic retina. This is mainly due to toxic products generated by the saturation of several physiological pathways related to glucose metabolism, including the polyol pathway, the hexosamine biosynthesis pathway, the formation of advanced glycation and lipoxidation end products, and protein kinase C (PKC) activation [1]. Histological sections from the retinas of vehicle-treated *Trpv2*^+/−^ rats showed higher levels of 8-hydroxyguanosine, indicating greater oxidative damage to DNA compared to wild-type control rats. Additionally, we observed for the first time that the retinas of this new model exhibited lower gene expression of key antioxidant enzymes like *Sod1*, *Sod2*, or *Gsr*, all essential for maintaining the physiological redox state. Furthermore, mRNA levels of the antioxidant transcription factor *Nrf2* were also increased, possibly as a defense mechanism to restore physiological levels of these enzymes, among others not studied [43, 44]. Topical administration of sitagliptin inhibited oxidative DNA damage and prevented the downregulation of *Sod1* and *Sod2* mRNA levels. This corroborates our findings in the *db/db* mouse model, with the exception that sitagliptin did not prevent the downregulation of *Gsr* mRNA levels in this case.

Regarding the vascular component of the NVU, we did not observe any visible alterations through fundus imaging among the different experimental groups. The presence of signs of neurodegeneration, such as retinal thinning and glial activation, despite the absence of early visible retinal damage (e.g., microaneurysms, hemorrhages, exudates, or cotton wool spots), confirms that by 3 months of age, the *Trpv2*^+/−^ rat model successfully recapitulates the early stages of DR observed in most patients. Using ARIA v1.0 software, we detected abnormal vasodilation of the main arterioles and venules of the retina in *Trpv2*^+/−^ rats, as occurs in DR. This anomaly was prevented with sitagliptin eye drops, with treated heterozygous animals exhibiting vessel diameters similar to those of the wild-type group. Although sitagliptin has been associated with both vasodilatory and vasoconstrictive effects [45, 46], to the best of our knowledge, there is no information on its effect in the retinal microcirculation. Our results suggest sitagliptin exerts a protective effect on the retinal NVU and prevents the abnormal vasodilation that occurs in *Trpv2*^+/−^ rats. This effect is significant when considering the pathophysiology of DR, as one consequence of diabetes induced NVU impairment is the lack of adaptation to metabolic demands, which could lead to capillary dropout. In fact, we have observed a significant loss of endothelial cells in the capillaries of *Trpv2*^+/−^ rats, which was prevented by topical administration of sitagliptin. This finding indicates that despite having a more direct effect on neurons, the drug is also capable of protecting the vascular elements of the NVU, thus confirming its dual action: neurotrophic and vasculotrophic.

The precise mechanisms by which *Trpv2*^+/−^ rats manifest the main features of early stages of DR are not fully understood. However, it should be noted that impaired regulation of blood flow precedes clinically observed lesions in the diabetic retina [37]. Therefore, the *Trpv2*^+/−^ model replicates the disease from one of its earliest pathological events: the loss of the myogenic response and failed autoregulation within the NVU. Oxidative stress, a major trigger of DR, is increased in this model despite the absence of hyperglycemia. A possible explanation is the initial impairment of neurovascular coupling due to a disrupted myogenic response, reducing the vasculature’s capacity to meet the metabolic demands of the retina, and altering physiological rates of oxygen delivery, consumption and ROS production [47]. Increased oxidative stress could trigger glial activation and inflammation, both of which also produce ROS, further worsening the process [48, 49]. Interestingly, we found that the retinal expression of glutamine synthetase (*Glul* gene) is not diminished in *Trpv2*^+/−^ rats. Glutamine synthetase, a key enzyme involved in glial-neuronal transmitter recycling and exclusively expressed by Müller cells within the retina, converts neurotransmitters substances [glutamate and gamma-aminobutyric acid (GABA)] into glutamine to maintain neurotransmitter balance and avoid glutamate excitotoxicity, a major pathogenic pathway of neuronal cell loss in DR [50]. This finding suggests that hyperglycaemia and/or its downstream metabolic pathways are crucial in the downregulation of glutamine synthetase observed in DR and that vascular hemodynamic changes secondary to the abnormal autoregulatory response do not affect this enzyme, at least in the early stages of DR.

**Fig. 9:**
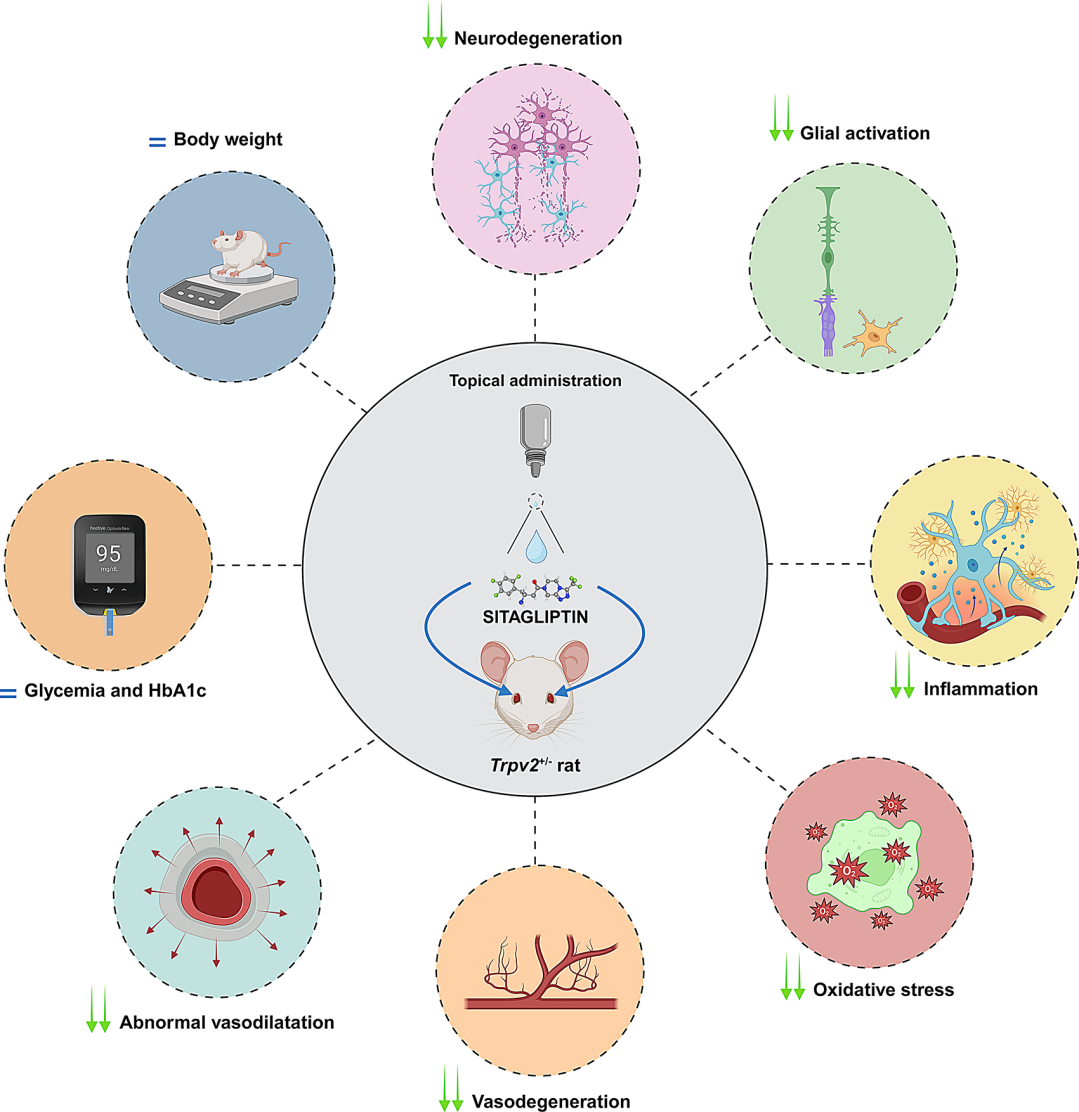
Primary effects of sitagliptin eye drops in the *Trpv2*^+/−^ model after two-weeks of twice-daily administration. Topical application of sitagliptin resulted in a reduction of neurodegeneration (preservation of total and inner retinal thickness), glial activation (reduction in GFAP-positive fibers and microglial cells), inflammation (decreased pro-inflammatory cytokines), oxidative stress (diminished DNA/RNA damage and maintenance of antioxidant elements), vasodegeneration (protection against the formation of acellular capillaries) and abnormal vasodilation. These effects were independent of changes in body weight, glycemia, and HbA1c levels

Overall, the similarity of retinal findings observed *Trpv2*^+/−^ rats to those observed in diabetic retinas indicates that the impairment of the autoregulation in the NVU could trigger the neurodegenerative processes present in DR. However, whether *Trpv2* is expressed in neurons and glial cells and its potential pathophysiological repercussions remains to be examined. Nevertheless, our results suggest that the *Trpv2*^+/−^ rat model, at 3 months of age, represents a good model for studying early stages of DR and for testing experimental preventive drugs against these stages of the disease. A graphical summary of the results obtained with sitagliptin eye drops is depicted in Fig. 9.

An important aspect of the results obtained is the potential translational value of the beneficial effects of sitagliptin eye drops for other diseases where NVU damage plays a key role in their pathophysiology. These diseases include glaucoma, retinitis pigmentosa, age-related macular degeneration, tauopathies, and even retinal damage associated with neurodegenerative diseases of the central nervous system, such as Alzheimer’s disease and Parkinson’s disease [51–54].

In conclusion, the beneficial effects of topical administration of sitagliptin, previously demonstrated in the *db/db* mouse model, are applicable to the non-diabetic *Trpv2*^+/−^ model, where DR-like lesions are not mediated by hyperglycemia. These findings suggest the potential utility of sitagliptin eye drops for treating early DR in humans and present an opportunity to explore its applicability in addressing other retinal diseases, particularly those characterized by NVU dysfunction and neurodegeneration.

## Data Availability

No datasets were generated or analysed during the current study.
